# Efficacy of a novel topical combination of esafoxolaner, eprinomectin and praziquantel in cats against *Toxocara cati* and *Dipylidium caninum*

**DOI:** 10.1051/parasite/2021024

**Published:** 2021-04-02

**Authors:** Martin Knaus, Christine Baker, Roberto Alva, Elizabeth Mitchell, Jennifer Irwin, Enstela Shukullari, Abdullah Veliu, Froylán Ibarra-Velarde, Julian Liebenberg, Craig Reinemeyer, Eric Tielemans, Kenneth Wakeland, Chris Johnson

**Affiliations:** 1 Boehringer Ingelheim Vetmedica GmbH, Kathrinenhof Research Center Walchenseestr. 8–12 83101 Rohrdorf Germany; 2 Boehringer Ingelheim Animal Health 1730 Olympic Drive Athens GA 30601 USA; 3 Faculty of Veterinary Medicine, Agricultural University of Tirana Rruga Paisi Vodica 1025 Tirana Albania; 4 Center for Animal Services, Fond. Prosp. Integr. Rr. Mine Peza, Pall. 3, Shk. 1/4 1000 Tirana Albania; 5 Universidad Nacional Autónoma de México, Ciudad Universitaria Coyoacan 04510 Mexico; 6 Clinvet International (Pty) Ltd. P.O. Box 11186, Universitas Bloemfontein 9321 Republic of South Africa; 7 East Tennessee Clinical Research 80 Copper Ridge Farm Rd Rockwood TN 37854 USA; 8 Boehringer-Ingelheim Animal Health 29 Avenue Tony Garnier 69007 Lyon France; 9 Boehringer-Ingelheim Animal Health 3239 Satellite Blvd Duluth GA 30096 USA

**Keywords:** Cat, Intestinal helminth, Esafoxolaner, Eprinomectin, Praziquantel, Efficacy

## Abstract

NexGard^®^ Combo, a novel topical antiparasitic product for cats, combines the insecticide/acaricide esafoxolaner with the nematocide eprinomectin and cestodicide praziquantel. The efficacy of this combination product was evaluated against two common endoparasites of global occurrence in cats, the nematode *Toxocara cati* and the cestode *Dipylidium caninum*, in five controlled studies using naturally or experimentally infected cats with parasites of North American, South African or European origin. Cats evaluated in these studies harbored patent infection of the target parasite confirmed through a pre-treatment fecal examination. In each study, cats were allocated randomly to two groups of equal size (8 or 10 cats per group per study), one group treated with a placebo (mineral oil) and the other with NexGard^®^ Combo. Both treatments were administered once as a spot-on at 0.12 mL per kg body weight to deliver the minimum label dosage (1.44 mg/kg esafoxolaner, 0.48 mg/kg eprinomectin, and 10.0 mg/kg praziquantel) to the NexGard^®^ Combo-treated cats. To determine efficacy, geometric mean parasite counts seven to 12 days after treatment of placebo-treated (control) cats and NexGard^®^ Combo-treated cats were compared. The efficacy of NexGard^®^ Combo was 98.8% and 100% against adult *T. cati* in two studies; and 98.0%, 98.3% and 93.2% against *D. caninum* in three studies*.* No adverse events related to treatment were observed throughout the studies. These studies demonstrate high efficacy against these major feline endoparasites and excellent acceptability of the novel topical antiparasitic combination of esafoxolaner, eprinomectin and praziquantel.

## Abstract (fr)

NexGard^®^ Combo, un nouveau produit antiparasitaire topique pour chats combine l’insecticide/acaricide esafoxolaner avec le nématocide éprinomectine et le cestodicide praziquantel. L’efficacité de ce produit d’association a été évaluée contre deux endoparasites communs d’occurrence mondiale chez le chat, le nématode *Toxocara cati* et le cestode *Dipylidium caninum*, dans cinq études contrôlées utilisant des chats naturellement ou expérimentalement infectés par des parasites d’origine nord-américaine, sud-africaine ou européenne. Les chats évalués dans ces études présentaient une infection patente du parasite cible confirmée par un examen fécal avant le traitement. Dans chaque étude, les chats ont été répartis au hasard en deux groupes de taille égale (8 ou 10 chats par groupe et par étude), un groupe traité avec un placebo (huile minérale) et l’autre avec NexGard^®^ Combo. Les deux traitements ont été administrés une fois par spot-on à 0,12 mL par kg de poids corporel pour délivrer la dose minimale indiquée sur l’étiquette (1,44 mg/kg d’esafoxolaner, 0,48 mg/kg d’éprinomectine et 10,0 mg/kg de praziquantel) pour les chats du groupe traité par NexGard^®^ Combo. Pour déterminer l’efficacité, les nombres moyens géométriques de parasites sept à 12 jours après le traitement des chats traités par placebo (témoins) et des chats traités par NexGard^®^ Combo ont été comparés. L’efficacité de NexGard^®^ Combo était de 98,8 % et de 100 % contre *T. cati* adulte dans deux études, et de 98,0 %, 98,3 % et 93,2 % contre *D. caninum* dans trois études. Aucun événement indésirable lié au traitement n’a été observé tout au long des études. Ces études démontrent la grande efficacité contre ces principaux endoparasites félins et l’excellente acceptabilité de la nouvelle combinaison antiparasitaire topique d’esafoxolaner, d’éprinomectine et de praziquantel.

## Introduction

Domestic cats may harbor a wide range of intestinal parasites, such as nematodes, cestodes, trematodes and protozoans [5]. The prevalence of intestinal helminths in domestic cats is variable and depends on several factors such as the cat’s age, its habitat (e.g., urban, rural), lifestyle habits (e.g., outdoor access, stray, shelter, domestic pet), and most fundamentally, its predatory behavior [2, 7, 28, 29, 34, 36, 39]. *Toxocara cati* is the most common nematode and the main ascarid species found in cats, with a high prevalence in kittens, while *Toxascaris leonina* and hookworms, such as *Ancylostoma tubaeforme*, *Ancylostoma braziliense* and *Ancylostoma ceylanicum* are more rarely diagnosed in domestic cats [5]. Major cestodes of domestic cats are dipylidiid cestodes such as *Dipylidium caninum*, *Joyeuxiella* spp. and *Diplopylidium* spp., and taeniid cestodes such as *Taenia taeniaeformis* and *Echinococcus multilocularis* [5].

Some of these helminths are also well known for the zoonotic risk to humans cohabiting with or exposed to infected cats. For example, *T. cati* may cause larva migrans in children as a result of oral ingestion of infective eggs [9, 10, 12, 17], and *D. caninum* may cause intestinal disorders in humans following flea ingestion [14, 18].

An effective deworming program is essential to control environmental contamination, thereby mitigating clinical implications in infected cats and minimizing the risk of spread and transmission to other felines and to people [11, 35].

NexGard^®^ Combo, a novel topical endectoparasiticide formulation for cats combines esafoxolaner, an isoxazoline with insecticidal and acaricidal activity, with two anthelminitic compounds of well-known efficacy, eprinomectin and praziquantel [8, 19–22, 32].

They have become standard therapeutics, as they have been proven safe and highly efficacious against a broad spectrum of intestinal helminths in cats [19–22, 32, 33, 35]. The marketed feline topical products Broadline™ and Centragard™ deliver the same dosage of eprinomectin and praziquantel as NexGard^®^ Combo, resulting in similar eprinomectin and praziquantel plasma profiles [16].

The studies reported here were conducted to confirm the efficacy and acceptability of this novel topical product in cats against naturally acquired and experimentally induced infections with *T. cati* or *D. caninum,* as per the requirements of regulatory agencies.

## Materials and methods

### Ethics

The study protocols were reviewed and approved by the Sponsor’s Institutional Animal Care and Use Committee and the studies were conducted according to local animal welfare legislation. Cats were handled with due regard for their wellbeing.

### Study design and study animals

The design of the studies was in accordance with the International Cooperation on Harmonisation of Technical Requirements for Registration of Veterinary Medicinal Products VICH GL7, “Efficacy of Anthelmintics: General Requirements” [37] and VICH GL20 “Efficacy of Anthelmintics: Specific Recommendations for Felines” [38], and the “World Association for the Advancement of Veterinary Parasitology (WAAVP) guidelines for evaluating the efficacy of anthelmintics for dogs and cats” [15]. All studies were conducted according to the principles of VICH GL9 entitled *Good Clinical Practice*. Personnel involved with the evaluation of efficacy and acceptability were unaware of the treatment assignments.

All studies used a randomized design for allocation of the cats to two groups, NexGard^®^ Combo-treated or placebo-treated (control). A total of 96 Domestic Short-hair cats, weighing between 1.0 and 5.0 kg prior to treatment, aged 3 months to 9 years and owned by the respective contract research organization were included in the studies. The studies were conducted in the United States (Study 1), Albania (Studies 2 and 4), South Africa (Study 3), and Mexico (Study 5). The animals were acclimated to the study facilities for at least seven days prior to treatment administration and cats were housed individually during the entire study duration. The environmental conditions were the same for all animals within a study. Study specific Information such as animal details, allocation, and target parasites are summarized in Table 1.

**Table 1:** Study-specific information: animal details, allocation, and target parasites.

	Study 1	Study 2	Study 3	Study 4	Study 5
Target parasite (origin)	*Toxocara cati* (Tennessee, USA)	*Toxocara cati* (Albania)	*Dipylidium caninum* (South Africa)	*Dipylidium caninum* (Albania)	*Dipylidium caninum* (Mexico)
Infection	Induced	Natural	Induced	Natural	Natural
Age of cats, range	19–21 weeks	5–8 months	1–9 years	2–4 years	3 months to 3 years
Bodyweight of cats, range (kg)	2.0–2.9	1.0–2.1	2.5–4.6	1.2–3.6	1.3–5.0
Number of cats per group	10	10	8	10	10
Allocation of cats to treatment groups	Completely at random	At random after blocking by bodyweight	At random after blocking by bodyweight	At random after blocking by bodyweight	At random after blocking by bodyweight
Timing of parasite recovery after treatment	7 days	7/8 days	7 days	10/11 days	10–12 days

Purpose-bred cats negative for patent infections with intestinal helminths were included in the two studies using experimentally induced infections (Studies 1 and 3). Study 1 cats were inoculated orally with approximately 34 larvated *T. cati* eggs daily from the same bulk solution on three consecutive days (63, 62 and 61 days before treatment). The parasite isolate originated from Tennessee, USA. The inoculation schedule was designed so that *T. cati* would be mature adult worms at the time of treatment. For Study 3, cats were experimentally infected with *D. caninum*, as described previously [3, 13], and monitored until patent infections were confirmed. The *D. caninum* isolate originated from a cat in South Africa.

Three studies involved naturally infected animals (Studies 2, 4 and 5) and used locally sourced cats that were selected based on positive fecal examination for the target parasite.

### Pre-treatment fecal examination

For all studies, the selection criterion for inclusion of cats was the presence of patent infection with the target parasite by examination of fecal samples collected within 9 days prior to the day of treatment, and all cats included were confirmed positive for at least fecal stages of the target parasite (Table 2). Fecal samples were examined macroscopically for the presence of cestode segments. In addition, feces were tested for helminth eggs using quantitative flotation techniques (Study 1, modified Wisconsin technique; Studies 2, 4 and 5, modified McMaster techniques).

**Table 2:** Results of pre-treatment fecal examinations.

	Study 1	Study 2	Study 3	Study 4	Study 5
Target parasite	*Toxocara cati*	*Toxocara cati*	*Dipylidium caninum*	*Dipylidium caninum*	*Dipylidium caninum*
Type of Infection	Induced	Natural	Induced	Natural	Natural
*Toxocara* eggs^a^					
Placebo (control)^1^	10/10	10/10	–	8/10	7/10
	(899–1944)	(500–5800)	–	(200–6350)	(350–4100)
Treated^2^	10/10	10/10	–	4/10	5/10
	(904–2292)	(100–5200)	–	(300–1550)	(400–6300)
Hookworm eggs^a^					
Placebo (control)	–	0/10	–	3/10	0/10
	–	–	–	(50–200)	–
Treated	–	2/10	–	0/10	1/10
	–	(0–1150)	–	–	(300)
Dipylidiid eggs/proglottids^b^					
Placebo (control)	–	0/10	8/8	10/10	10/10
Treated	–	0/10	8/8	10/10	10/10

a Number of cats testing positive/number of cats in group; (range of eggs per gram counts).

b Number of cats testing positive/number of cats in group.

1 Mineral oil at 0.12 mL per kg bodyweight spot-on, once.

2 NexGard^®^ Combo at 0.12 mL per kg bodyweight spot-on, once.

### Treatment administration

All cats were treated once on Day 0. Cats assigned to the placebo (control) group were administered mineral oil topically at 0.12 mL per kg bodyweight. Cats assigned to the NexGard^®^ Combo (esafoxolaner 1.2% w/v, eprinomectin 0.4% w/v, praziquantel 8.3% w/v) group received a topical (spot-on) application of the formulation at the minimum recommended dosage of 0.12 mL/kg body weight, delivering 1.44 mg esafoxolaner, 0.48 mg eprinomectin, and 10.0 mg praziquantel per kg body weight. The treatment was applied directly on the skin, after parting the hair, in one spot in the midline of the neck between the base of the skull and the shoulder blades.

### Helminth recovery and count

Animals were humanely euthanized following AVMP Guidelines [1] and necropsied 7–12 days after treatment administration for parasite recovery and count. The contents of the whole gastrointestinal tract (stomach, small and large intestines) including scraped mucosa were washed over appropriately sized sieves to remove debris, and examined for helminths. Worm counts were made on total gastrointestinal contents. Helminths were identified to species/genus, and stage, as appropriate, according to their morphology. The number of scolices was counted for cestode numeration.

### Acceptability of treatment and health

Health observations were conducted daily throughout the studies by qualified personnel and at hourly intervals for 4 h after treatment to detect any health abnormalities.

### Statistical analysis

For each study, target parasite counts were transformed to the natural logarithm of (count +1) for calculation of geometric means for the treatment group. Efficacy was determined for the target parasite by calculating the percent efficacy as 100[(*C* − *T*)/*C*], where *C* was the geometric mean among controls, and *T* was the geometric mean among the treated animals. The log-counts of both groups were compared using an F-test adjusted for the allocation blocks used to randomize the animals to the treatment groups (Studies 2–5). The MIXED procedure in SAS was used for the analysis, with the treatment groups listed as a fixed effect and the allocation blocks listed as a random effect. For Study 1 the log-counts of both groups were compared using the GLM Procedure in SAS. All testing was two-sided at the significance level *α* = 0.05.

## Results

No adverse events or other health problems related to treatment with NexGard^®^ Combo were observed throughout the studies.

The results (parasite counts, percentage efficacy, statistical comparison of treatment groups) of the five studies for the target parasites are summarized in Table 3. Cats treated with NexGard^®^ Combo had significantly fewer adult *T. cati* ascarids and *D. caninum* tapeworms than the placebo-treated controls. In the individual studies, the percentage efficacies against adult *T. cati* were 98.8% and 100%, and against *D. caninum* were 93.2%, 98.0% and 98.3%. All studies were considered to have adequate challenge based on target parasite infections in the cats of at least five adult *T. cati* or at least two *D. caninum* recovered from a minimum of six cats of the respective placebo-treated control groups.

**Table 3:** Parasite counts and efficacy.

Parasite	Study/treatment groups	Number of positive cats/number of cats in group	Geometric mean parasite count (range)	Efficacy^1^ (%)	*p*-value^2^
Adult *Toxocara cati*	Study 1, induced infection (Tennessee, USA)				
	Placebo (control)^3^	10/10	29.5 (14–57)	100	<0.0001
	Treated^4^	0/10	0		
	Study 2, natural infection (Albania)				
	Placebo (control)	10/10	12.1 (2–32)	98.8	<0.0001
	Treated	1/10	0.1 (0–3)		
*Dipylidium caninum*	Study 3, induced infection (South Africa)				
	Placebo (control)	7/8	28.9 (8–267)	98.0	0.0018
	Treated	2/8	0.6 (3–9)		
	Study 4, natural infection (Albania)				
	Placebo (control)	9/10	23.1 (3–143)	98.3	0.0001
	Treated	2/10	0.4 (1–12)		
	Study 5, natural infection (Mexico)				
	Placebo (control)	7/10	5.6 (1–370)	93.2	0.0052
	Treated	1/10	0.4 (24)		

1 Percent efficacy = 100[(*C* − *T*)/*C*], where *C* was the geometric mean among placebo controls and *T* was the geometric mean among the treated animals.

2 Two-sided probability value from analysis of variance on log-counts of the treated group and the placebo control group.

3 Mineral oil at 0.12 mL per kg bodyweight spot-on, once.

4 NexGard^®^ Combo at 0.12 mL per kg bodyweight spot-on, once.

Inoculation with approximately 100 larvated *T. cati* eggs per cat in Study 1 resulted in the recovery of 319 adult *T. cati* in the placebo-treated controls, indicating a mean rate of establishment of 31.9%.

In the three studies using naturally infected animals, non-target parasites were recovered from one to nine cats in the placebo-treated control groups, including adult *Ancylostoma tubaeforme* (Studies 2 and 4), fourth-stage *T. cati* (Study 2), adult *T. cati* (Studies 4 and 5), dipylidiid cestodes (*D. caninum*, *Diplopylidium* spp. and *Joyeuxiella pasqualei*; Studies 2 and 4) and/or *Taenia taeniaeformis* (Study 5). However, the number of parasites recovered was not considered meaningful for efficacy calculations because the presence of patent infection with these parasites had not been demonstrated prior to treatment in a minimum of six cats in each of the treatment groups (Table 2).

## Discussion

The results of the present studies testing the efficacy of the novel topical product NexGard^®^ Combo administered to cats demonstrate that eprinomectin and praziquantel in combination with esafoxolaner provide high efficacy against adult *T. cati* and *D. caninum* infections. One administration of NexGard^®^ Combo at the minimum label dose resulted in greater than 98% reduction of adult *T. cati* ascarid burden, and greater than 93% reduction of *D. caninum* tapeworm burden.

These results are consistent with the level of efficacy in a series of controlled studies assessing treatment with Broadline^TM^, which delivers the same eprinomectin and praziquantel dosage as NexGard^®^ Combo. These studies on experimentally and naturally infected cats demonstrated efficacy against adult *T. cati* and *D. caninum* ranging from 97.1% to 100% and 97.7% to 99.2%, respectively [19, 20]. Results of the controlled laboratory Broadline^TM^ studies were also supported in a multi-center field efficacy study demonstrating 99.9% and 100% efficacy against *T. cati* and *D. caninum*, respectively, based on results of examination of feces [32]. Equivalence of efficacy (100%) for the praziquantel component delivered by the treatment of cats with the minimum label dose of Broadline^TM^ and NexGard^®^ Combo was also demonstrated against *Echinococcus multilocularis* cestode infections [30, 35]. The results of the present studies demonstrate the biologically equivalent anthelmintic efficacy against three parasites of both eprinomectin and praziquantel in NexGard^®^ Combo and Broadline^TM^. The effect of these compounds is not impacted by the presence of the isoxazoline compound esafoxolaner in the formulation, resulting in eprinomectin and praziquantel plasma profiles that are considered to be similar to those of the above products [16].

The mode of inoculation in studies that involved experimentally infected cats performed well. The counts of adult *T. cati* recovered from the placebo-treated control cats in Study 1 provide further support for experimental regimens using lower doses of larvated eggs over two to three consecutive days, rather than a single larger dose at once, as previously recommended [20]. The rate of establishment and numbers of *D. caninum* in the placebo-treated control cats in Study 3 confirmed the suitability of the inoculation model, as previously described [3, 4, 13]. The spectrum of helminths recovered from the placebo-treated cats in the studies with naturally infected animals (Studies 2, 4 and 5) confirms their presence in domestic cats in the countries where the studies were conducted [6, 15] and re-emphasises the multi-species nature of feline intestinal parasitism. Diagnosis of cestode infections in cats requires special attention and may not be successful using routine methods. None of the cats in Study 2 were found to shed fecal forms of dipylidiid cestodes at a single fecal examination prior to treatment. However, 9 out of 10 placebo control cats were found with specimens of *D. caninum*. This supports the hypothesis that cestode infections are largely underestimated in cats [27].

The equivalent levels of efficacy demonstrated against adult *T. cati* and *D. caninum,* which were considered the least sensitive nematode and cestode species for eprinomectin and praziquantel, respectively, may reasonably allow to conclude that the administration of NexGard^®^ Combo to cats will provide the same range of activity as does the administration of Broadline^TM^ with respect to nematode and cestode infections. Thus, the spectrum of activity of NexGard^®^ Combo in cats may comprise migrating and intestinal larval *T. cati*, adult *Toxascaris leonina*, adult and fourth-stage *A. braziliense, A. ceylanicum* and *A. tubaeforme* hookworms, adult and larval *Aelurostrongylus abstrusus* and *Troglostrongylus brevior* lungworms, vesical and pulmonary *Capillaria* (*C. plica, C. aerophila*) and *Diplopylidium* spp., and *J. pasqualei* and *T. taeniaeformis* cestodes [19–26, 31–33] in addition to adult *T. cati*, *D. caninum* and *E. multilocularis*.

## Conclusion

The results of the present series of studies demonstrated that NexGard^®^ Combo is safe and highly effective against experimentally induced and naturally acquired infections with adult *T. cati* ascarids and *D. caninum* tapeworms in cats. The product can therefore be assumed to provide efficacious and convenient treatment against a broad range of intestinal nematodes and cestodes parasitizing domestic cats worldwide.

## Competing interest

The work reported herein was funded by Boehringer-Ingelheim Animal Health. The authors are current employees of Boehringer-Ingelheim or external contractors. Other than that, the authors declare no conflict of interest. This document is provided for scientific purposes only. Any reference to a brand or trademark herein is for information purposes only and is not intended for any commercial purposes or to dilute the rights of the respective owners of the brand(s) or trademark(s). Broadline™, Centragard^®^ and NexGard^®^ are registered trademarks of Boehringer-Ingelheim Group.
